# GZ17-6.02 kills PDX isolates of uveal melanoma

**DOI:** 10.18632/oncotarget.28586

**Published:** 2024-05-17

**Authors:** Laurence Booth, Jane L. Roberts, Ivan Spasojevic, Kaitlyn C. Baker, Andrew Poklepovic, Cameron West, John M. Kirkwood, Paul Dent

**Affiliations:** ^1^Department of Biochemistry and Molecular Biology, Virginia Commonwealth University, Richmond, VA 23298, USA; ^2^Department of Medicine, Virginia Commonwealth University, Richmond, VA 23298, USA; ^3^Department of Medicine, and PK/PD Core Laboratory, Duke University School of Medicine, Durham, NC 27710, USA; ^4^Genzada Pharmaceuticals, Hutchinson, KS 67502, USA; ^5^Department of Dermatology, Texas Tech University Health Sciences Center, Lubbock, TX 79430, USA; ^6^Melanoma and Skin Cancer Program, Hillman Cancer Research Pavilion Laboratory, University of Pittsburgh Cancer Institute, Pittsburgh, PA 15213, USA

**Keywords:** autophagy, ER stress, GZ17-6.02, doxorubicin, afatinib, neratinib

## Abstract

GZ17-6.02 has undergone phase I evaluation in patients with solid tumors (NCT03775525). The RP2D is 375 mg PO BID, with an uveal melanoma patient exhibiting a 15% reduction in tumor mass for 5 months at this dose. Studies in this manuscript have defined the biology of GZ17-6.02 in PDX isolates of uveal melanoma cells. GZ17-6.02 killed uveal melanoma cells through multiple convergent signals including enhanced ATM-AMPK-mTORC1 activity, inactivation of YAP/TAZ and inactivation of eIF2α. GZ17-6.02 significantly enhanced the expression of BAP1, predictive to reduce metastasis, and reduced the levels of ERBB family RTKs, predicted to reduce growth. GZ17-6.02 interacted with doxorubicin or ERBB family inhibitors to significantly enhance tumor cell killing which was associated with greater levels of autophagosome formation and autophagic flux. Knock down of Beclin1, ATG5 or eIF2α were more protective than knock down of ATM, AMPKα, CD95 or FADD, however, over-expression of FLIP-s provided greater protection compared to knock down of CD95 or FADD. Expression of activated forms of mTOR and STAT3 significantly reduced tumor cell killing. GZ17-6.02 reduced the expression of PD-L1 in uveal melanoma cells to a similar extent as observed in cutaneous melanoma cells whereas it was less effective at enhancing the levels of MHCA. The components of GZ17-6.02 were detected in tumors using a syngeneic tumor model. Our data support future testing GZ17-6.02 in uveal melanoma as a single agent, in combination with ERBB family inhibitors, in combination with cytotoxic drugs, or with an anti-PD1 immunotherapy.

## INTRODUCTION

GZ17-6.02 is a novel compound, containing the synthetically manufactured components: curcumin, harmine and isovanillin and has undergone phase I safety evaluation in cancer patients (NCT03775525). The recommended phase 2 dose (RP2D) is 375 mg PO BID, with an uveal melanoma patient exhibiting a 15% reduction in tumor mass for 5 months at this dose. We have published data showing that GZ17-6.02 kills a diverse range of tumor cell types, including prostate, ER+ breast, colorectal, pancreatic, hepatic, biliary, NSCLC, cutaneous melanoma, sarcoma and actinic keratoses [1–9].

Uveal melanoma (UM) is an uncommon cancer of the eye with an incidence of approximately 1 person per 100,000 of population [10]. Recently, the FDA approved the drug Kimmtrak (Tebentafusp) for a specific small subset of UM patients expressing the marker HLA-A*02:01, however for the majority of patients, there remains no good therapeutic intervention [11]. In approximately 90% of UM patients have driving mutations in G alpha proteins that, like mutant RAS proteins in other tumor types, have lost their GTPase activity [12–16]. In approximately 50% of UM patients, BRCA1 associated protein-1, BAP1, (ubiquitin carboxy-terminal hydrolase), a deubiquitinating enzyme, is mutated inactive, i.e., BAP1 is a tumor suppressor, and its loss of function subsequently was also associated with BAP1 acting as a suppressor of metastatic spread [17–20]. BAP1 is proposed to regulate the amount of ubiquitination of histones, regulating homeobox genes and long-term cell fate and stem-cell like behavior [21, 22].

GZ17-6.02 simultaneously regulates multiple cell signaling processes which converge to kill tumor cells. Activation of ataxia telangiectasia (ATM) alongside reduced signaling by receptor tyrosine kinases which resulted in the inactivation of mTORC1 and that was responsible for enhanced autophagosome formation and autophagic flux. PKR-like endoplasmic reticulum kinase (PERK) or PKR based on the cell type were activated concomitant with increased the phosphorylation (inactivation) of eIF2α [1–9]. This acted to both reduce the protein levels of protective MCL-1 and BCL-XL and to enhance expression of the autophagy-regulatory proteins Beclin1 and ATG5. Based on the tumor cell type, GZ17-6.02 also in a cell-type dependent fashion enhanced death receptor signaling, with activation of caspase 8 and cleavage of the toxic BH3 domain protein BID leading to activation of BAX and BAK [1–9].

In animal models of colon and prostate cancer, GZ17-6.02 as a single agent significantly prolonged animal survival and interacted with 5-fluorouracil in the colon cancer cells to further enhance survival [7]. In LNCaP prostate cancer tumors treated for 45 days with vehicle control or GZ17-6.02, all control animals had died prior to day 45 whereas for animals treated with GZ17-6.02 the median survival was 78 days, i.e., tumor growth control was maintained for ~33 days in the absence of drug [8]. A phase Ib trial is planned in hormone refractory prostate cancer at Massey Cancer Center.

We have previously published that PDX isolates of UM were sensitive to irreversible inhibitors of ERBB family members, particularly the multi-kinase inhibitor neratinib [23]. Neratinib enhanced autophagosome formation and could reduce receptor expression. Others have shown that neratinib can enhance macroautophagy and reduce receptor expression [24, 25]. The present studies defined the biology of GZ17-6.02 in UM cells and in parallel determined its interaction with irreversible ERBB inhibitors (afatinib, neratinib) and with the cytotoxic agent doxorubicin.

## RESULTS

**Figure 1:**
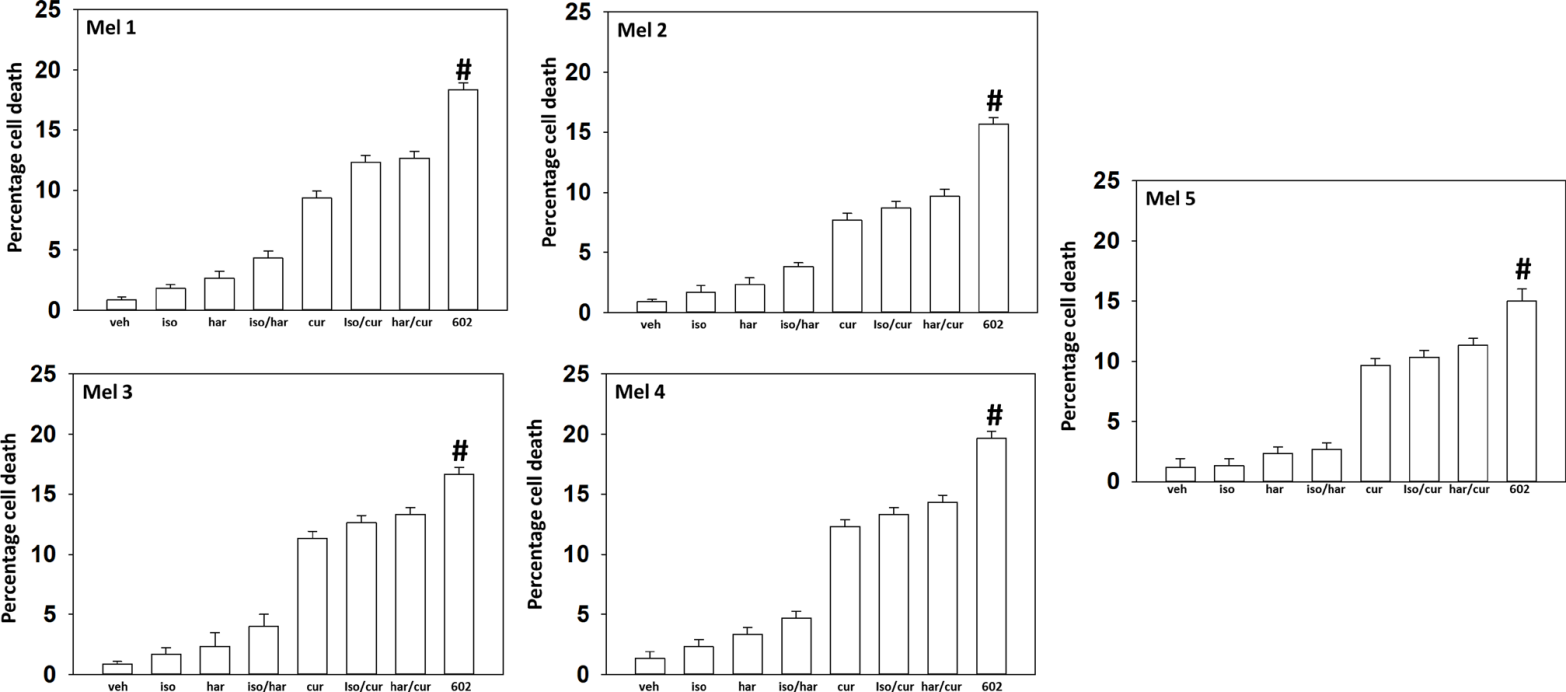
GZ17-6.02 kills uveal melanoma cells more efficaciously than the individual components. PDX isolates of uveal melanoma were treated with vehicle control, GZ17-6.02 (curcumin (2.0 μM) + harmine (4.5 μM) + isovanillin (37.2 μM)) or with component parts of GZ17-6.02 as individual agents at the indicated concentrations or in duo combinations. Cells were isolated 24 h afterwards and viability determined via trypan blue exclusion assays (*n* = 3 +/− SD). ^#^ *p* < 0.05 greater than other tested drug treatments.

**Figure 2:**
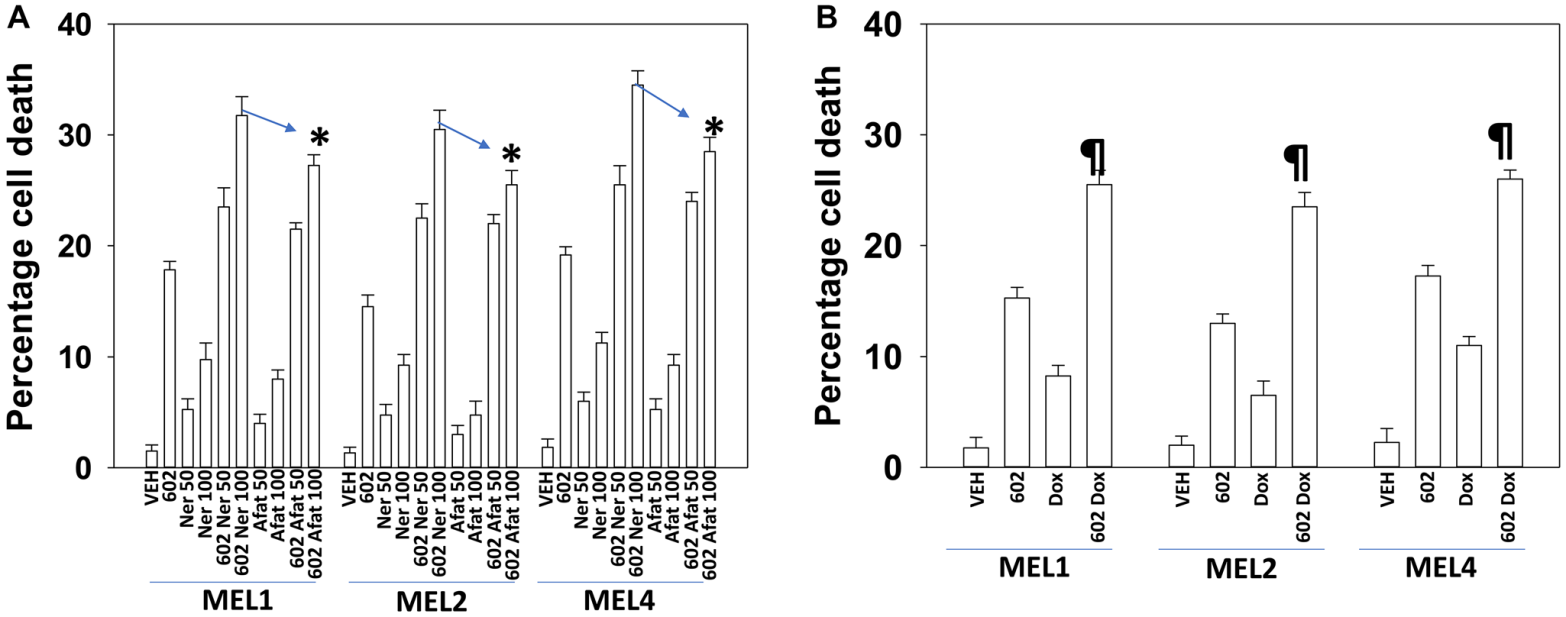
GZ17-6.02 kills uveal melanoma cells more efficaciously than the individual components. (**A**) PDX isolates of uveal melanoma were treated with vehicle control, GZ17-6.02 (curcumin (2.0 μM) + harmine (4.5 μM) + isovanillin (37.2 μM)), afatinib (50 nM, 100 nM) or neratinib (50 nM, 100 nM) alone or in the indicated combinations. Cells were isolated 24 h afterwards and viability determined via trypan blue exclusion assays (*n* = 3 +/− SD). ^*^ *p* < 0.05 less than the corresponding value in afatinib treated cells. (**B**) Uveal melanoma cells were treated with vehicle control, GZ17-6.02 (2 μM, curcumin, final concentration), doxorubicin (50 μM) or in combination for 24 h. Cells were isolated 24 h afterwards and viability determined via trypan blue exclusion assays (*n* = 3 +/− SD). ^¶^ *p* < 0.05 less than the corresponding value in cells treated with 100 nM of the ERBB kinase inhibitors.

GZ17-6.02 comprises by mass three chemically synthesized (pure) natural products: curcumin (10%); harmine (13%); isovanillin (77%). Compared to its individual component parts of harmine, isovanillin and curcumin, as single agents or together in pairs, the three-compound GZ17-6.02 was the most efficacious agent at killing UM cells (Figure 1). Afatinib, neratinib and doxorubicin interacted with GZ17-6.02 in an additive fashion to further enhance UM cell killing. The combinatorial lethality of (afatinib plus 602) and (neratinib plus 602) were identical when the kinase inhibitors were used at a concentration of 50 nM, whereas at a concentration of 100 nM, still below the C max of the drugs in patient plasma, neratinib was slightly more efficacious than afatinib (Figure 2A). The combination of GZ17-6.02 and doxorubicin exhibited less killing than the combination of GZ17-6.02 with neratinib or afatinib (Figure 2B).

The recommended phase 2 dose (RP2D) of GZ17-6.02 is 375 mg PO BID. A syngeneic mouse model of uveal melanoma is not commercially available. Using a comparable low amount of GZ17-6.02, 50 mg/kg, dosing once daily, we determined the uptake of curcumin, harmine and isovanillin in established CT26 mouse colorectal tumors growing in their immune-competent syngeneic BALB/c mouse host. UM tumors express mutant G alpha proteins whereas the CT26 line expresses a mutated GTPase inactive KRAS protein. After thirty consecutive days of GZ17-6.02 dosing, tumors were isolated and processed to determine the concentrations of curcumin, harmine and isovanillin in the tumors (Table 1). All three components of GZ17-6.02 were detected. As was observed previously in prostate cancer studies, mice dosed with GZ17-6.02 did not lose body mass (data not shown) [8].

**Table 1:** Treatment of animals with GZ17-6.02 results in tumors containing all three components of the drug: curcumin, harmine and isovanillin

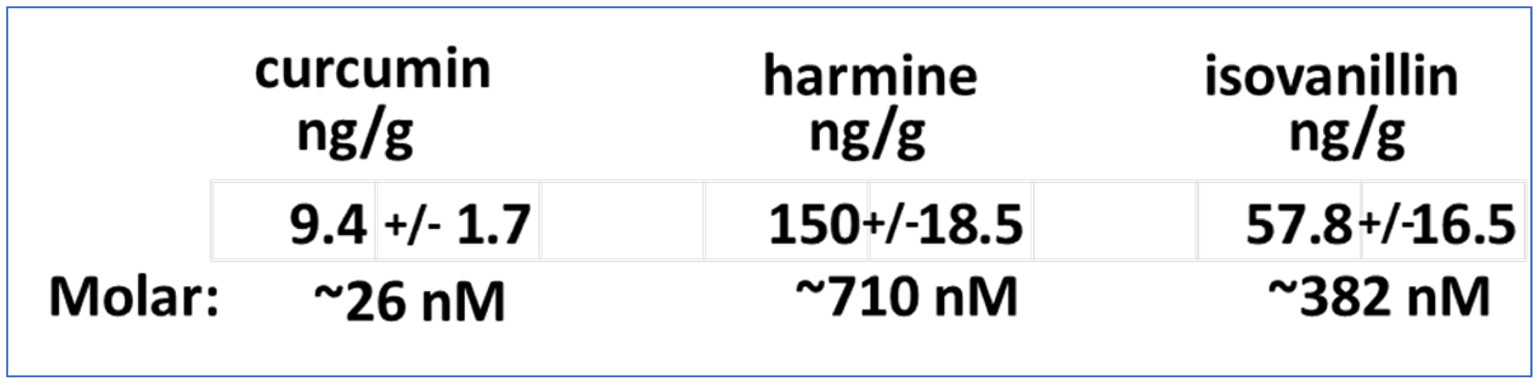

Immune-competent BALB/c mice were implanted with syngeneic CT26 cells (1 × 10^6^) and tumors were permitted to grow for 14 days until they reached a volume of ~100 mm^3^. Control mice (5 animals) were treated daily by gavage with vehicle control. Mice (10 animals) were treated daily by gavage with GZ17-6.02 (50 mg/kg). Mice were treated for thirty days. Tumors were then isolated, and flash frozen in liquid N_2_. Tumor materials were processed to determine their levels of curcumin, harmine and isovanillin as described in the Methods section. Control tumors did not contain GZ17-6.02 (not shown). (*n **=*** 10 independent treated mice/tumors +/− SD).

We next defined changes in cell signaling processes when we combined GZ17-6.02 with afatinib, neratinib or doxorubicin. GZ17-6.02 interacted with afatinib, neratinib and doxorubicin to activate ATM and the AMPK and inactivate mTORC1 (Tables 2 and 3). GZ17-6.02 interacted with neratinib to inactivate AKT, p70 S6K, ERK1/2, STAT3, STAT5, NFΚB, c-SRC, eIF2α. Neratinib and GZ17-6.02 interacted in both tested lines to inactivate ERBB3. Downstream of these signaling events we observed enhanced expression of Beclin1 and ATG5 and increased phosphorylation of ATG13, which predicts we could observe autophagosome formation.

**Table 2:** Regulation of cell signaling by GZ17-6.02, afatinib and neratinib in MEL1 uveal melanoma cells

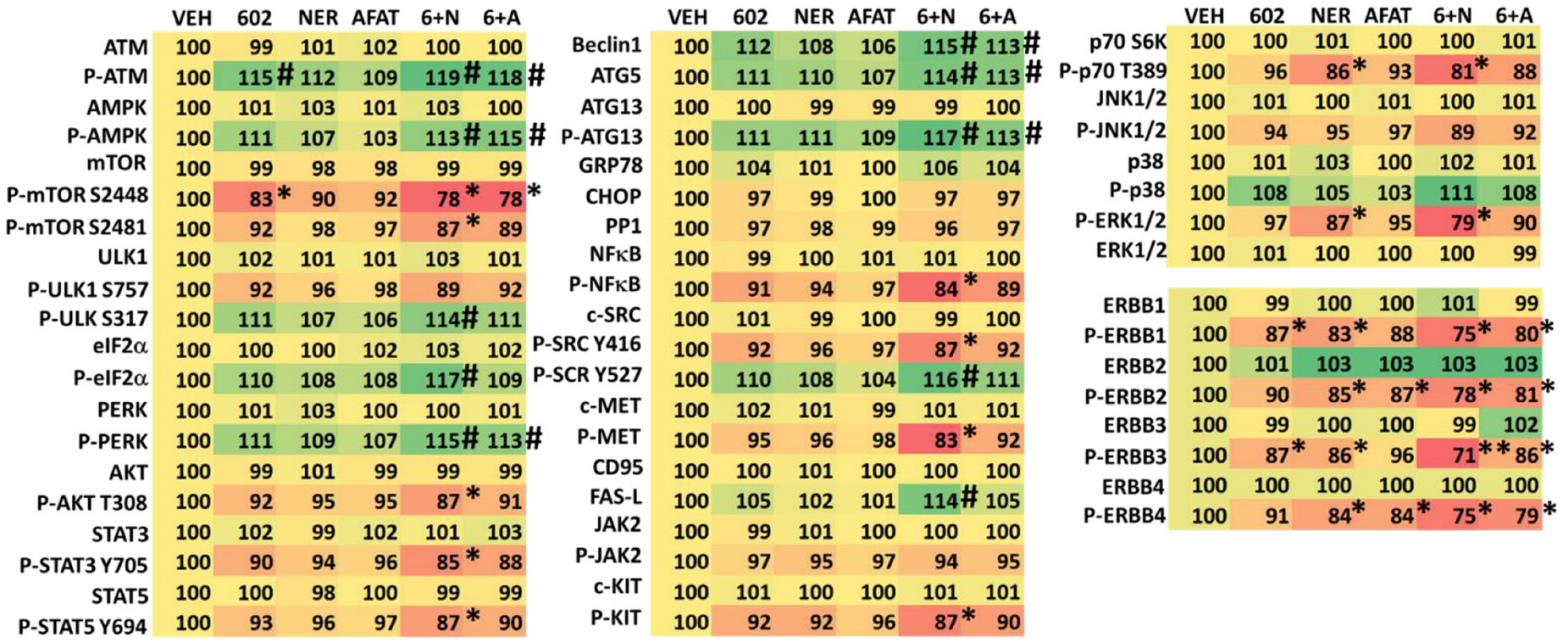

MEL1 cells were treated with vehicle control, GZ17-6.02 (2 μM, curcumin final), afatinib (100 nM), neratinib (100 nM) or the drugs in combination for 4 h. Cells were fixed *in situ*, permeabilized, stained with the indicated validated primary antibodies and imaged with secondary antibodies carrying red- and green-fluorescent tags. The staining intensity of at least 100 cells per well/condition is determined in three separate studies. The data are the normalized amount of fluorescence set at 100% comparing intensity values for vehicle control (*n **=*** 3 +/− SD). ^#^ *p* < 0.05 greater than vehicle control; ^*^ *p* < 0.05 less than vehicle control; ^**^ *p* < 0.05 less than GZ17-6.02 as a single agent.

**Table 3:** Regulation of cell signaling by GZ17-6.02, afatinib and neratinib in MEL4 uveal melanoma cells

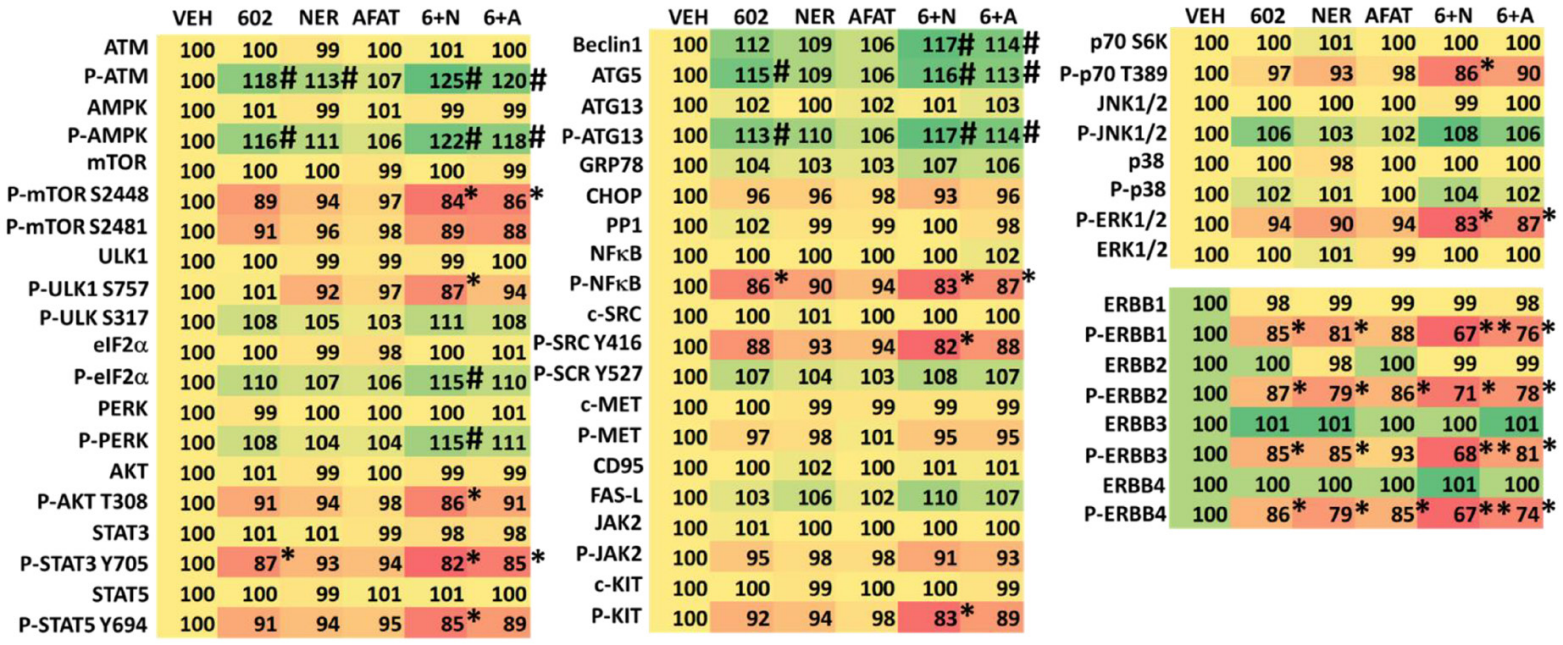

MEL4 cells were treated with vehicle control, GZ17-6.02 (2 μM, curcumin final), afatinib (100 nM), neratinib (100 nM) or the drugs in combination for 4 h. Cells were fixed *in situ*, permeabilized, stained with the indicated validated primary antibodies and imaged with secondary antibodies carrying red- and green-fluorescent tags. The staining intensity of at least 100 cells per well/condition is determined in three separate studies. The data are the normalized amount of fluorescence set at 100% comparing intensity values for vehicle control (*n **=*** 3 +/− SD). ^#^ *p* < 0.05 greater than vehicle control; ^*^ *p* < 0.05 less than vehicle control; ^**^ *p* < 0.05 less than GZ17-6.02 as a single agent.

**Figure 3:**
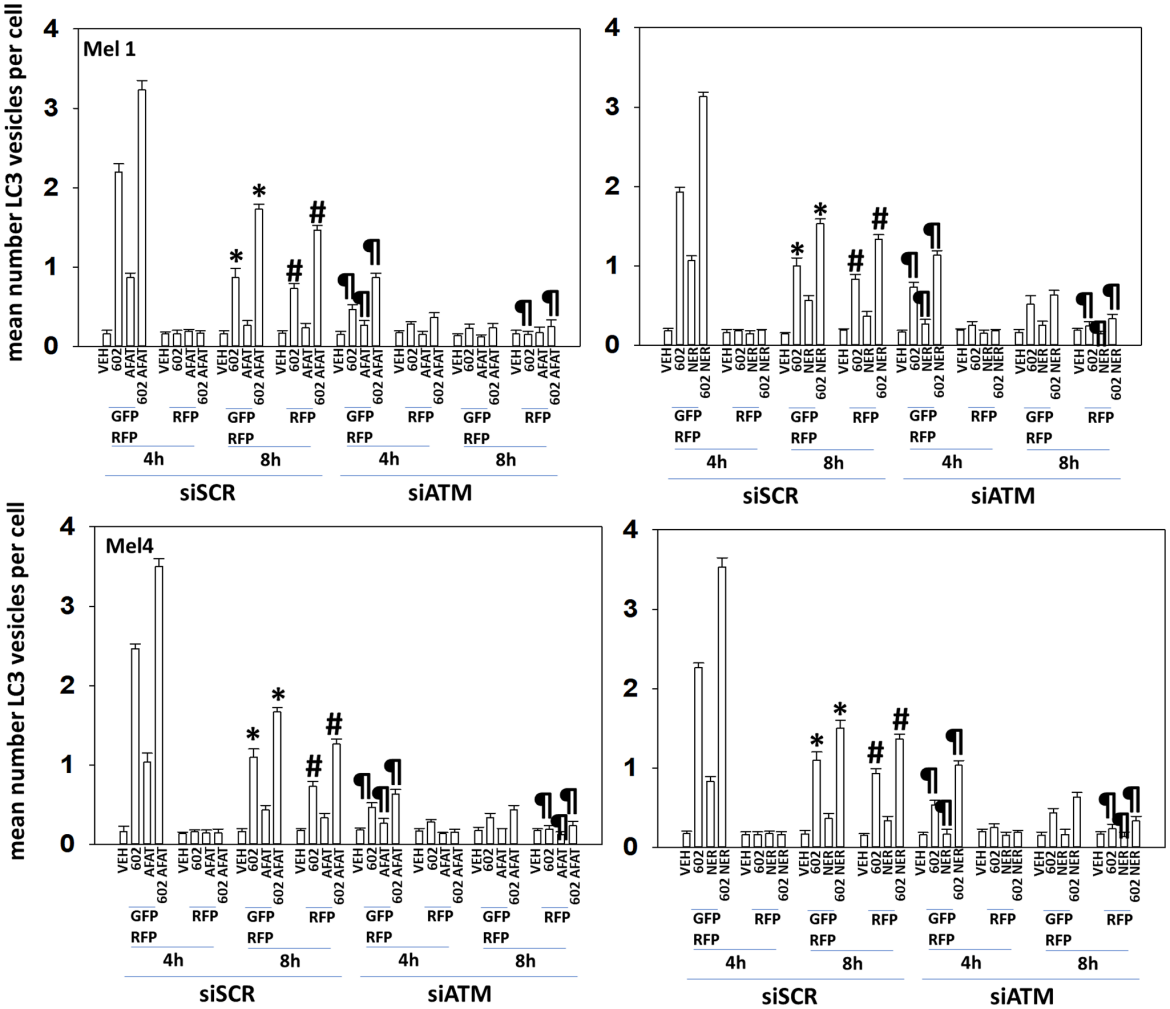
Regulation of macroautophagy by (GZ17-6.02 + ERBB inhibitors) requires ATM. Cells were transfected with a scrambled siRNA or with an siRNA to knock down expression of ATM. In parallel, cells were transfected with a plasmid to express LC3-GFP-RFP. After 24 h, cells were treated with vehicle control, GZ17-6.02 (2 μM, curcumin final), afatinib (100 nM), neratinib (100 nM) or in combination for 4 h and 8 h. At each time point, the mean number of GFP+RFP+ vesicles and only RFP+ vesicles per cell were determined in forty randomly selected cells (*n* = 3 +/− SD). ^*^ *p* < 0.05 less than corresponding values at 4 h; ^#^ *p* < 0.05 greater than corresponding values at 4 h; ^¶^ *p* < 0.05 less than corresponding values in siSCR cells.

Hence, we next examined autophagosome formation and autophagic flux using a plasmid to express LC3-GFP-RFP: autophagosomes stain (GFP+ RFP+) and acidic autolysosomes where GFP is quenched stain (RFP+). GZ17-6.02 interacted with both afatinib and neratinib to increase autophagosome formation which was temporally followed by a decrease in autophagosome numbers and an increase in autolysosome levels, i.e., autophagic flux (Figure 3). Knock-down of ATM significantly reduced the abilities of the drugs to cause formation of autophagosomes and autolysosomes.

**Figure 4:**
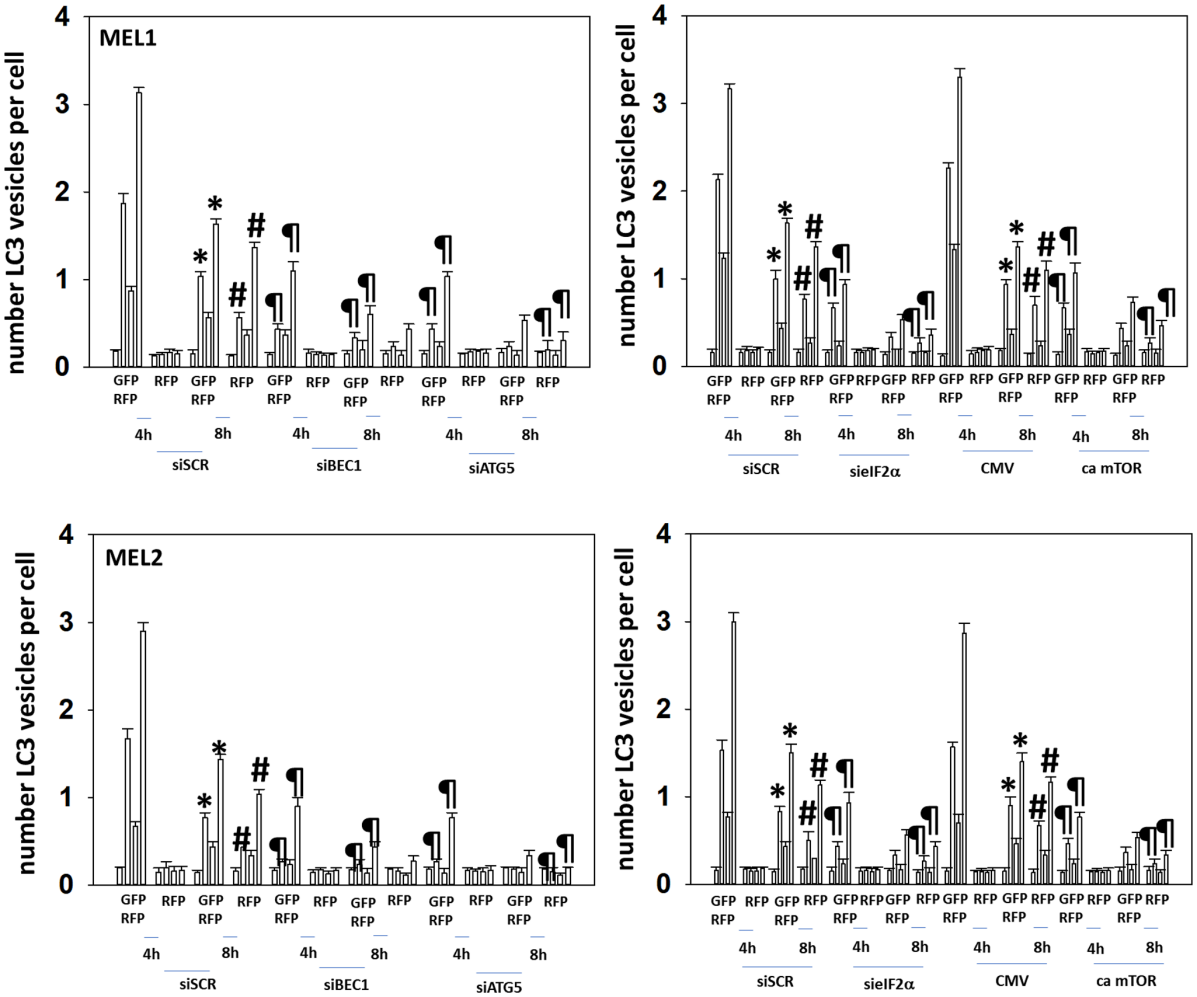
Knock down of Beclin1, ATG5 or eIF2α, or expression of an activated mTOR protein, reduces autophagosome formation and autophagic flux. Cells were transfected with a plasmid to express LC3-GFP-RFP and in parallel transfected with a scrambled siRNA or with siRNA molecules to knock down the expression of Beclin1, ATG5 or of eIF2α. In parallel, other cells were transfected with an empty vector plasmid (CMV) or a plasmid to express an activated form of mTOR. Twenty-four h later, cells were treated with vehicle control, GZ17-6.02 (2 μM, curcumin final), afatinib (100 nM), neratinib (100 nM) or the drugs in combination for 4 h and 8 h. At each time point, the mean number of GFP+RFP+ vesicles and only RFP+ vesicles per cell were determined in forty randomly selected cells (*n* = 3 +/− SD). ^*^ *p* < 0.05 less than corresponding values at 4 h; ^#^ *p* < 0.05 greater than corresponding values at 4 h; ^¶^ *p* < 0.05 less than corresponding values in siSCR cells.

**Figure 5:**
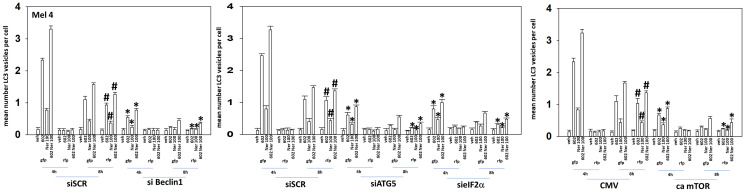
Knock down of Beclin1, ATG5 or eIF2α, or expression of an activated mTOR protein, reduces autophagosome formation and autophagic flux. MEL4 cells were transfected with a plasmid to express LC3-GFP-RFP and in parallel transfected with a scrambled siRNA or with siRNA molecules to knock down the expression of Beclin1, ATG5 or of eIF2α. In parallel, other cells were transfected with an empty vector plasmid (CMV) or a plasmid to express an activated form of mTOR. Twenty-four h later, cells were treated with vehicle control, GZ17-6.02 (2 μM), afatinib (100 nM), neratinib (100 nM) or the drugs in combination for 4 h and 8 h. At each time point, the mean number of GFP+RFP+ vesicles and only RFP+ vesicles per cell were determined in forty randomly selected cells (*n* = 3 +/− SD). ^*^ *p* < 0.05 less than corresponding values at 4 h; ^#^ *p* < 0.05 greater than corresponding values at 4 h.

We next determined the importance of Beclin1, ATG5, eIF2α and mTORC1 inactivation in the autophagy response when combining GZ17-6.02 and neratinib. Knock down of Beclin1, ATG5 or eIF2α significantly reduced the formation of autophagosomes and autolysosomes (Figures 4 and 5). Expression of an activated form of mTOR also significantly lowered the numbers of autophagosomes formed and reduced autolysosome formation. Collectively, the findings in Tables 2, 3, and Figures 3–5, link drug-induced changes in signaling to the regulation of macroautophagy in PDX uveal melanoma cells.

**Figure 6:**
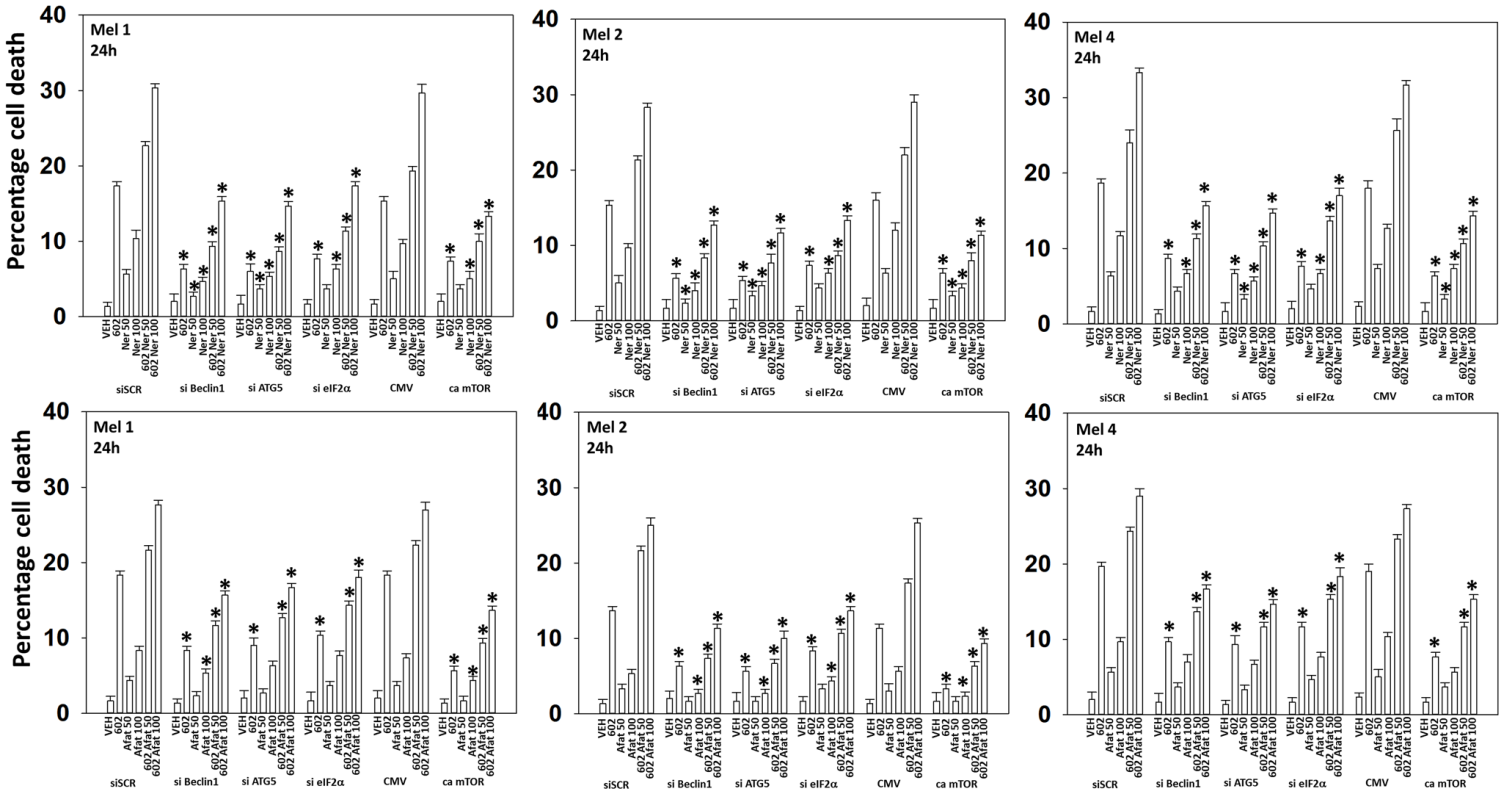
Cell-killing by (GZ17-6.02 + ERBB inhibitors) requires Beclin1, ATG5 and eIF2α, and is reduced by expression of activated mTOR. Cells were transfected with a scrambled siRNA or with an siRNA to knock down expression of Beclin1, ATG5 or eIF2α. In parallel, other cells were transfected with an empty vector plasmid (CMV) or a plasmid to express an activated form of mTOR. Twenty-four h later, cells were treated with vehicle control, GZ17-6.02 (2 μM, curcumin final), afatinib (50 nM, 100 nM), neratinib (50 nM, 100 nM) or the drugs in combination for 24 h. Cells were isolated 24 h afterwards and viability determined via trypan blue exclusion assays (*n* = 3 +/− SD). ^*^ *p* < 0.05 less than the corresponding value in cells transfected with scrambled siRNA or empty vector plasmid.

**Figure 7:**
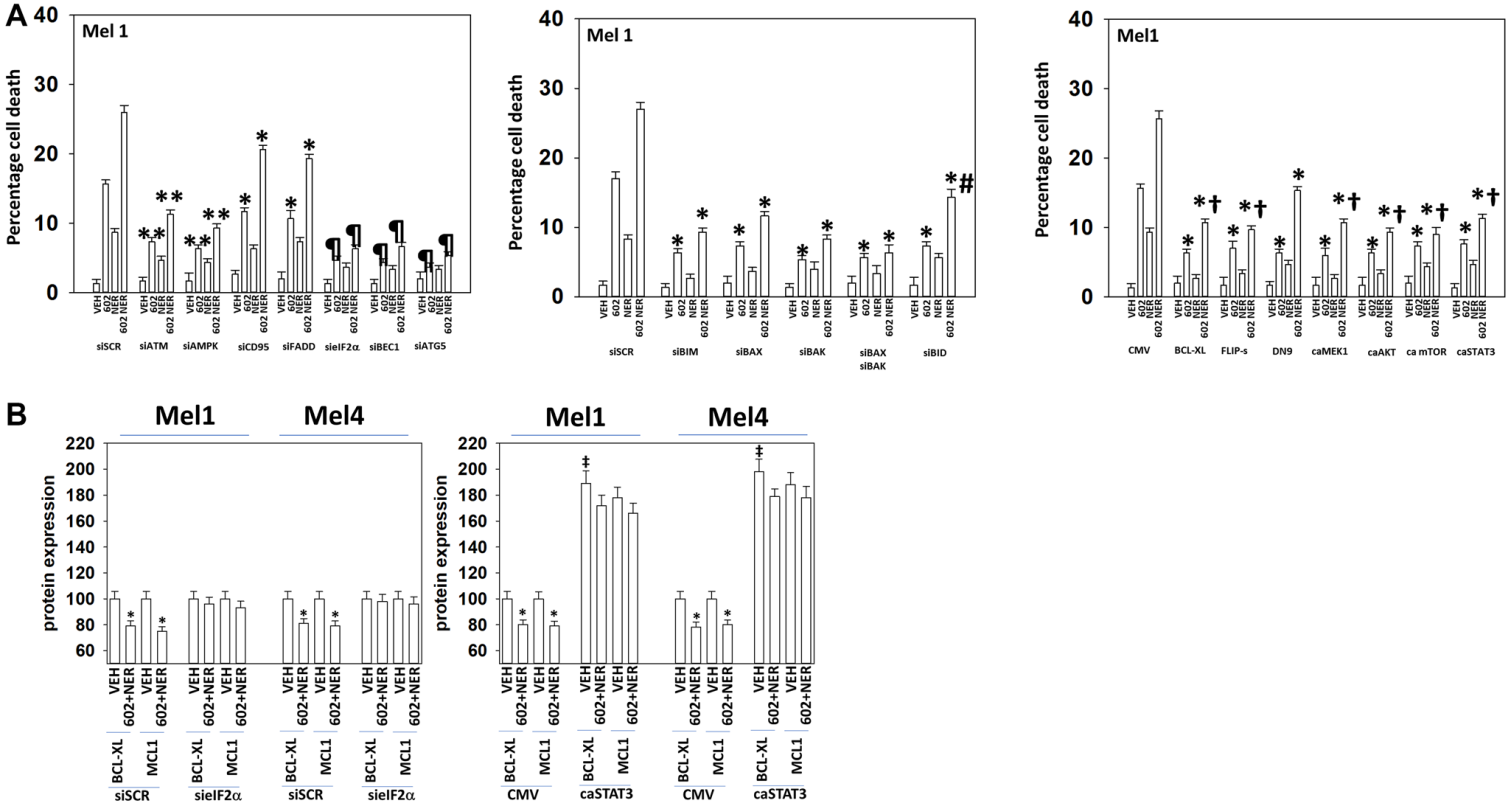
Drug lethality requires signaling by ATM-AMPK and the actions of toxic BH3 domain proteins. (**A**) Cells were transfected with a scrambled siRNA or with siRNA molecules to knock down the expression of ATM, AMPKα, CD95, FADD, eIF2α, Beclin1, ATG5, BIM, BAX, BAK or BID. In other portions of cells, they were transfected with an empty vector plasmid or with plasmids to express BCL-XL, FLIP-s, dominant negative caspase 9, activated MEK1, activated AKT, activated mTOR or activated STAT3. Twenty-four h later, cells were treated with vehicle control, GZ17-6.02 (2 μM, curcumin final), neratinib (100 nM), or the drugs in combination for 24 h. Cells were isolated 24 h afterwards and viability determined via trypan blue exclusion assays (*n* = 3 +/− SD). ^*^ *p* < 0.05 less than the corresponding value in cells transfected with scrambled siRNA or empty vector plasmid; ^**^ *p* < 0.05 less than corresponding values in siCD95 and siFADD cells; ^¶^ *p* < 0.05 less than corresponding values in siATM and siAMPKα; ^†^ *p* < 0.05 less than corresponding value in dominant negative caspase 9 expressing cells. (**B**) Mel1 and Mel4 cells were either transfected with a scrambled siRNA or with an siRNA to knock down eIF2α expression or transfected with an empty vector plasmid or with a plasmid to express activated STAT3. Twenty-four h later, cells were treated with vehicle control, GZ17-6.02 (2 μM, curcumin final), neratinib (100 nM), or the drugs in combination for 4 h. Cells were fixed in place and the expression of BCL-XL, MCL1 and ERK2 (not shown, loading control) determined. (*n* = 3 +/− SD) ^*^ *p* < 0.05 less than the corresponding value in cells transfected with scrambled siRNA or empty vector plasmid; ^‡^ *p* < 0.05 greater than corresponding value in CMV cells.

**Figure 8:**
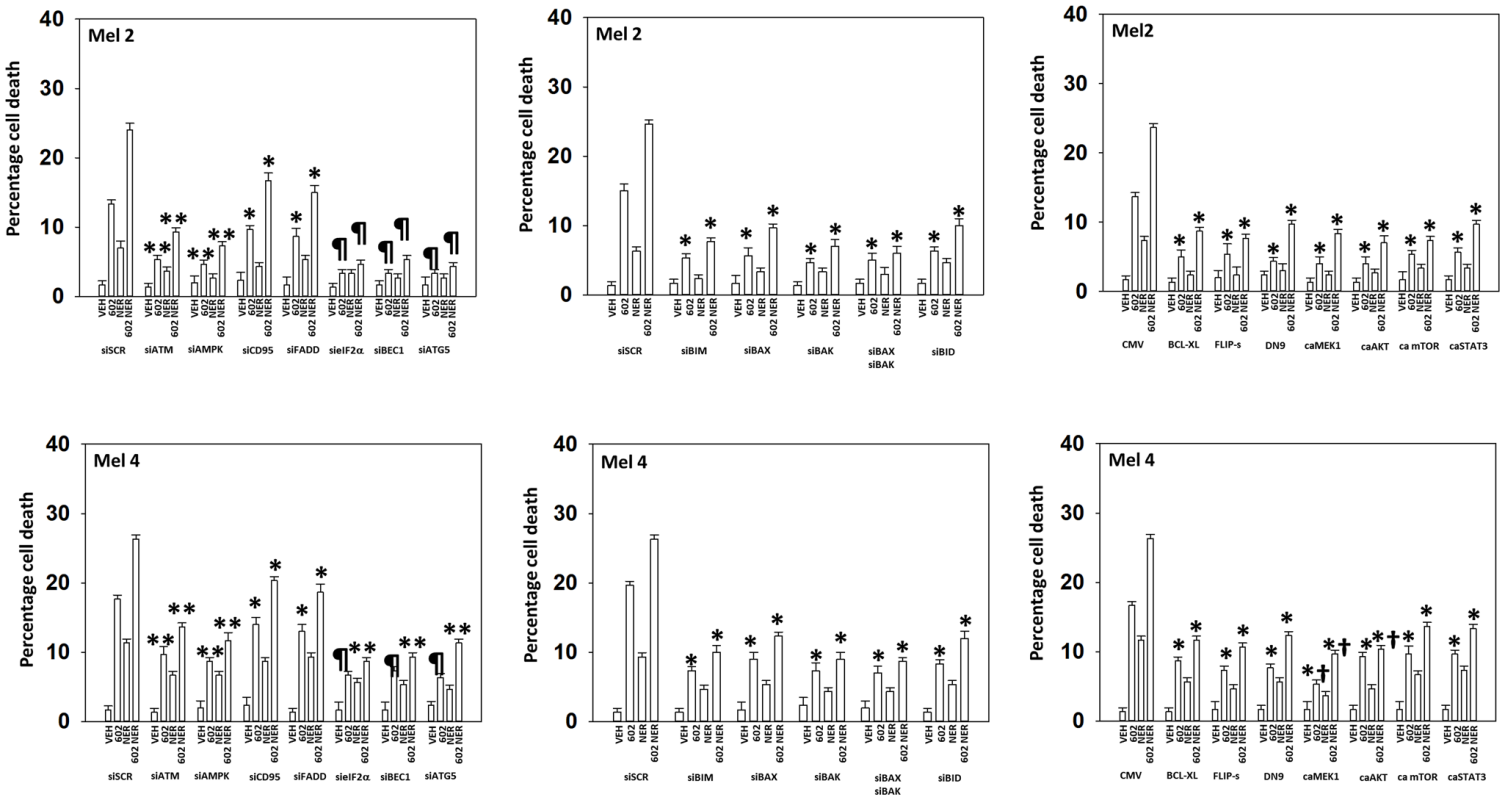
In MEL2 and MEL4 cells drug lethality requires signaling by ATM-AMPK and the actions of toxic BH3 domain proteins. Cells were transfected with a scrambled siRNA or with siRNA molecules to knock down the expression of ATM, AMPKα, CD95, FADD, eIF2α, Beclin1, ATG5, BIM, BAX, BAK or BID. In other portions of cells, they were transfected with an empty vector plasmid or with plasmids to express BCL-XL, FLIP-s, dominant negative caspase 9, activated MEK1, activated AKT, activated mTOR or activated STAT3. Twenty-four h later, cells were treated with vehicle control, GZ17-6.02 (2 μM), neratinib (100 nM), or the drugs in combination for 24 h. Cells were isolated 24 h afterwards and viability determined via trypan blue exclusion assays (*n* = 3 +/− SD). ^*^ *p* < 0.05 less than the corresponding value in cells transfected with scrambled siRNA or empty vector plasmid; ^**^ *p* < 0.05 less than corresponding values in siCD95 and siFADD cells; ^¶^ *p* < 0.05 less than corresponding values in siATM and siAMPKα; ^†^ *p* < 0.05 less than corresponding value in dominant negative caspase 9 expressing cells.

We next defined the role of macroautophagy and other survival-regulatory processes in the control of cell viability after drug exposure. Knock down of Beclin1, ATG5 or eIF2α significantly reduced the abilities of GZ17-6.02, afatinib and neratinib as single agents or when in combination to kill UM cells (Figure 6). Knock down of CD95 or of FADD was protective, but not to the same extent as knock down of ATM, AMPKα, eIF2α, Beclin1 or ATG5 (Figures 7A, 8). Over-expression of FLIP-s was protective in all three lines tested, significantly more protective than knock down of CD95/FADD, collectively arguing that caspase 8 was being activated via a feed-back loop with caspase 3, rather than from death receptor signaling. In MEL4 cells, the expression of activated MEK1 was significantly more protective than expression of activated mTOR or activated STAT3 (Figure 8). Treatment of UM cells with (GZ17-6.02 + neratinib) reduced the expression of BCL-XL and MCL1 (Figures 7B, 8). Knock down of eIF2α did not alter basal levels of BCL-XL or MCL1 but prevented the drug combination from reducing their expression. Expression of activated STAT3 increased basal expression of BCL-XL and MCL1 approximately 2-fold, and activated STAT3 also prevented the drug-induced decline in their protein levels. Thus, both ER stress signaling and reduced STAT3 signaling play overlapping roles in regulating MCL1 and BCL-XL levels that regulates tumor cell viability after drug exposure.

The Hippo pathway plays a key role in initiation, the development and therapeutic resistance in uveal melanoma [26–30]. Hence, we next defined the impact of GZ17-6.02, afatinib and neratinib on the Hippo pathway co-transcription factors YAP and TAZ. As single agents, both GZ17-6.02 and neratinib variably increased the phosphorylation of YAP S127 and YAP S397, whereas afatinib had no effect (Table 4). As single agents, both GZ17-6.02 and neratinib increased the phosphorylation of TAZ S89 whereas afatinib had no effect. When GZ17-6.02 was combined with neratinib, the phosphorylation of YAP S109, YAP S127 and YAP S397 was significantly elevated. Afatinib also interacted variably with GZ17-6.02 to enhance YAP phosphorylation in the MEL4 isolate. This data strongly supports the use of both GZ17-6.02 and neratinib as therapeutic agents to inactivate Hippo pathway signaling and suppress the growth and viability of uveal melanoma cells.

**Table 4:** Regulation of Hippo pathway signaling by GZ17-6.02, afatinib and neratinib in uveal melanoma cells

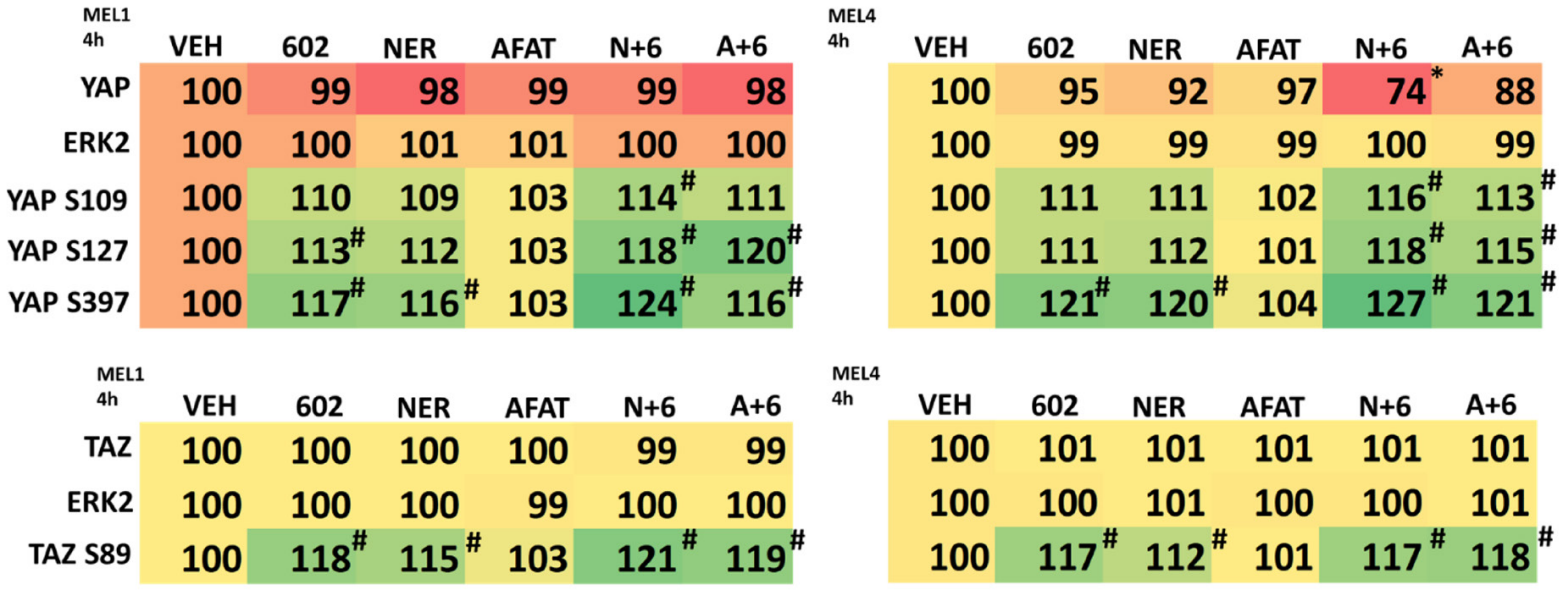

MEL1 and MEL4 cells were treated with vehicle control, GZ17-6.02 (2 μM, curcumin final), afatinib (100 nM), neratinib (100 nM) or the drugs in combination for 4 h. Cells were fixed *in situ*, permeabilized, stained with the indicated validated primary antibodies and imaged with secondary antibodies carrying red- and green-fluorescent tags. The staining intensity of at least 100 cells per well/condition is determined in three separate studies. The data are the normalized amount of fluorescence set at 100% comparing intensity values for vehicle control (*n **=*** 3 +/− SD). ^#^ *p* < 0.05 greater than vehicle control; ^*^ *p* < 0.05 less than vehicle control.

Unlike cutaneous melanoma, which responds to checkpoint inhibitory antibodies, uveal melanoma is considered to be “cold” to checkpoint inhibitory immunotherapy. We determined the effect of GZ17-6.02 exposure on the expression levels of checkpoint immunotherapy biomarkers PD-L1 and MHCA in uveal melanoma cells and compared the effects to those observed previously in other tumor cell types. GZ17-6.02 significantly reduced the expression of PD-L1 and enhanced the expression of MHCA (Table 5). However, these alterations in protein expression trended to be less than observed in many other tumor cell types, and for some tumor types this was significant. For example, the reductions in PD-L1 expression in NSCLC cells were significantly greater than those observed in the uveal melanoma cells. Similarly, the increases in MHCA expression in PDX isolates of cutaneous melanoma were significantly greater than was observed in uveal melanoma cells.

**Table 5:** GZ17-6.02 regulates the expression of PD-L1 and MHCA in uveal melanoma cells

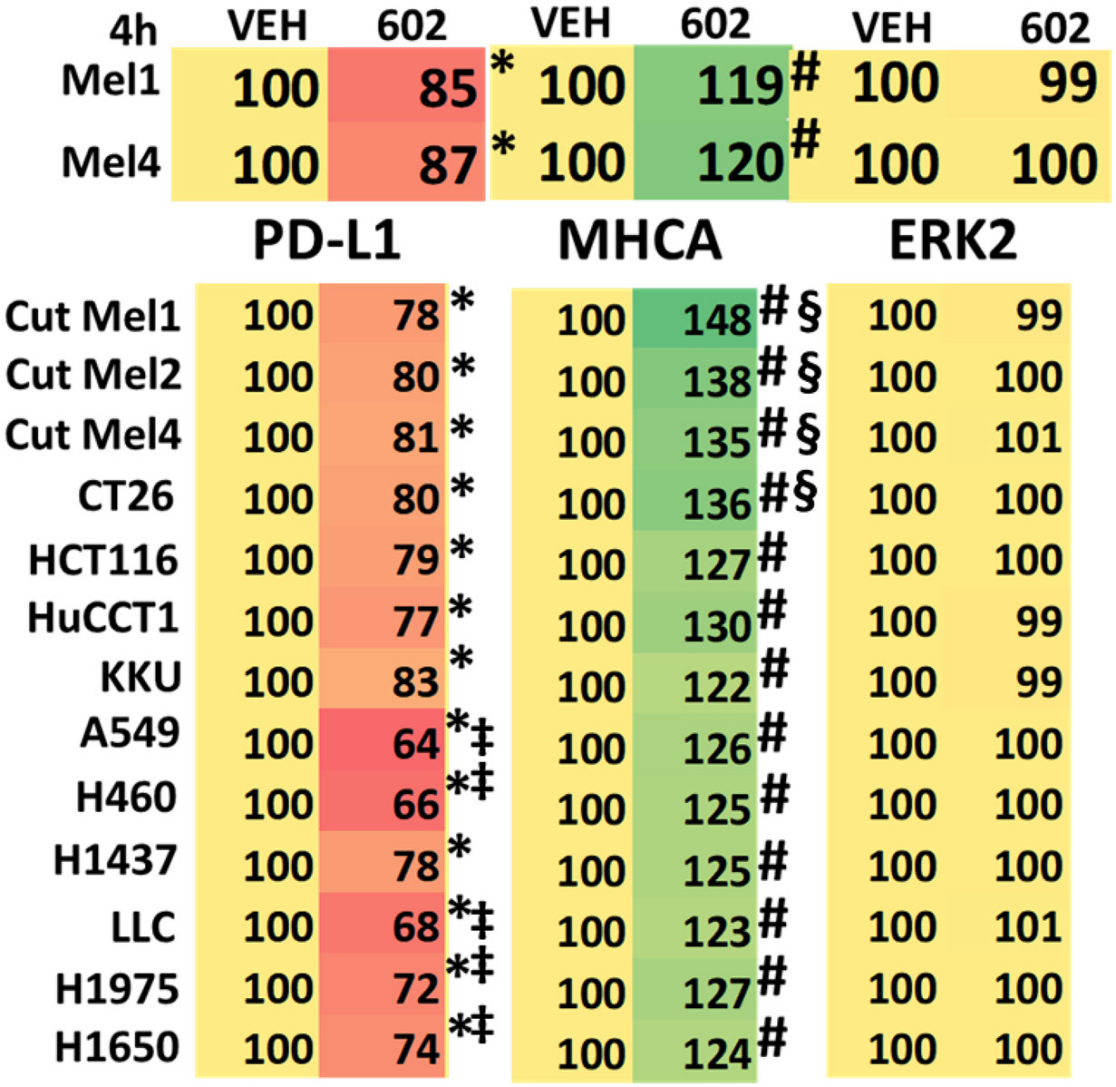

MEL1 and MEL4 cells were treated with vehicle control or GZ17-6.02 (2 μM, curcumin final) for 4 h. In parallel, other tumor cell types were also treated with GZ17-6.02. Cells were fixed *in situ*, permeabilized, stained with the indicated validated primary antibodies and imaged with secondary antibodies carrying red- and green-fluorescent tags. The staining intensity of at least 100 cells per well/condition is determined in three separate studies. The data are the normalized amount of fluorescence set at 100% comparing intensity values for vehicle control (*n **=*** 3 +/− SD). ^#^ *p* < 0.05 greater than vehicle control; ^*^ *p* < 0.05 less than vehicle control; ^‡^ *p* < 0.05 less than corresponding values in uveal melanoma cells; ^§^ *p* < 0.05 greater than corresponding values in uveal melanoma cells.

BAP1 (BRCA1 associated protein-1) a ubiquitin carboxy-terminal hydrolase is a tumor suppressor and prevents metastatic spread [31]. Approximately 50% of all uveal melanomas express a mutated inactive form of BAP1, with the majority of metastatic disease having BAP1 mutations [31–33]. Persons with a single germline mutant allele of BAP1 are also pre-disposed to developing uveal melanoma [34]. Thus, we determined the impact, if any, of GZ17-6.02, afatinib and neratinib upon the expression of BAP1 in our uveal melanoma isolates. As a single agent, GZ17-6.02 significantly enhanced BAP1 expression (Table 6). Neither afatinib nor neratinib altered BAP1 expression and they did not interact with GZ17-6.02 to further enhance BAP1 expression. Mutation of BAP1 can also result in alterations to histone methylation, and we further explored whether GZ17-6.02 changed the methylation of Histone H3 in uveal melanoma cells. Twenty-four h after treatment, the methylation of lysine 9 in histone H3 was reduced (Table 7). The methylation of lysine 4 was also reduced and this correlated with a trend for increased di-methylation of lysine 4. A trend was also observed for reduced lysine 27 di-methylation and lysine 79 methylation.

**Table 6:** GZ17-6.02 enhances BAP1 expression in uveal melanoma cells

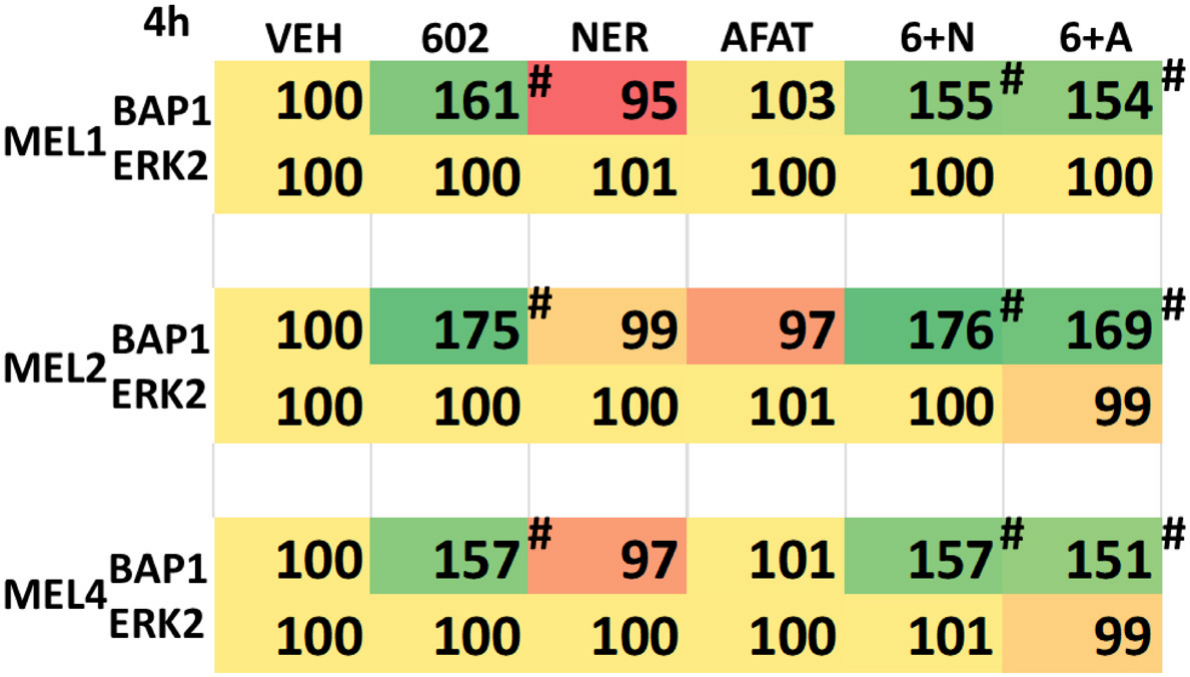

MEL1, MEL2 and MEL4 cells were treated with vehicle control, GZ17-6.02 (2 μM, curcumin final), afatinib (100 nM), neratinib (100 nM) or the drugs in combination for 4 h. Cells were fixed *in situ*, permeabilized, stained with the indicated validated primary antibodies and imaged with secondary antibodies carrying red- and green-fluorescent tags. The staining intensity of at least 100 cells per well/condition is determined in three separate studies. The data are the normalized amount of fluorescence set at 100% comparing intensity values for vehicle control (*n **=*** 3 +/− SD). ^#^ *p* < 0.05 greater than vehicle control.

**Table 7:** GZ17-6.02 and neratinib regulate Histone H3 methylation and acetylation in uveal melanoma cells

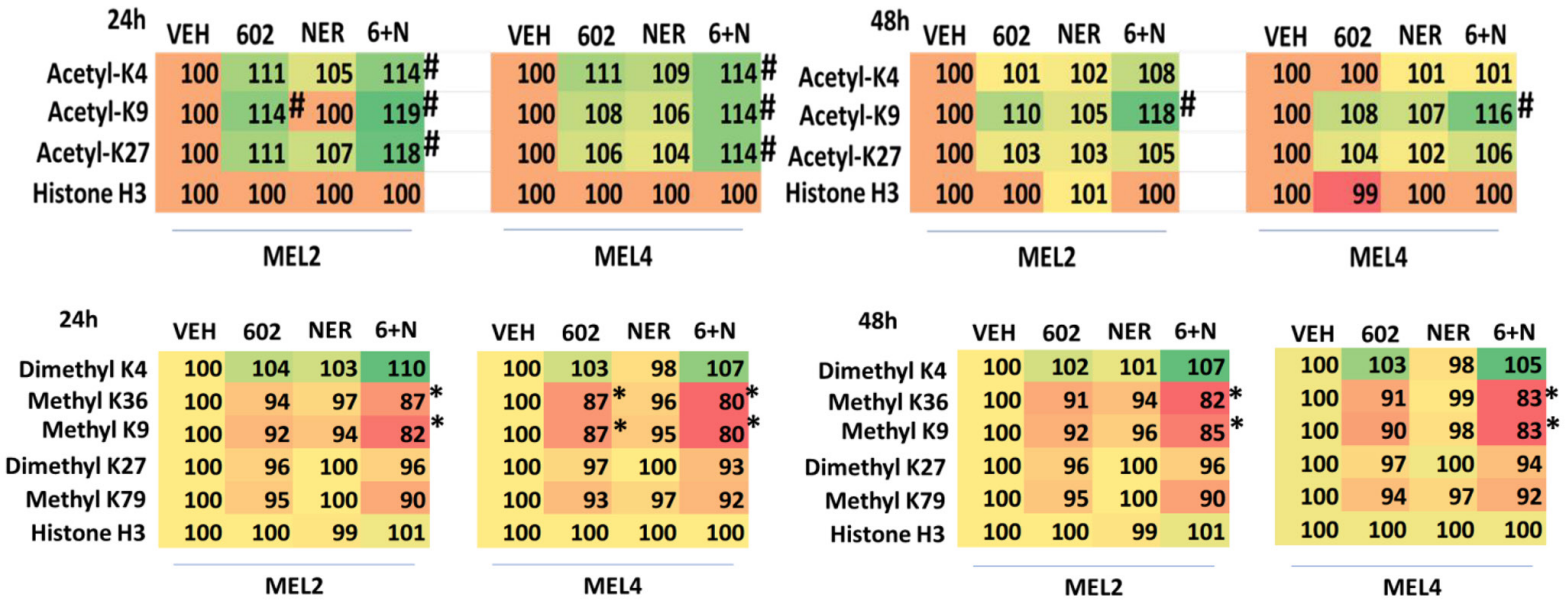

Cells were treated with vehicle control, GZ17-6.02 (2 μM, curcumin final), neratinib (100 nM) or the drugs in combination for 24 h. Cells were fixed *in situ*, permeabilized, stained with the indicated validated primary antibodies and imaged with secondary antibodies carrying red- and green-fluorescent tags. The staining intensity of at least 100 cells per well/condition is determined in three separate studies. The data are the normalized amount of fluorescence set at 100% comparing intensity values for vehicle control (*n **=*** 3 +/− SD). ^#^ *p* < 0.05 greater than vehicle control.

## DISCUSSION

The present studies demonstrated that GZ17-6.02 interacted with irreversible inhibitors of the EGF receptor and HER2 to kill uveal melanoma cells. The mechanisms by which the GZ17-6.02 interacted with the kinase inhibitors to cause tumor cell death were multi-factorial. GZ17-6.02 interacted with the kinase inhibitors to increase autophagosome formation and promote autophagic flux, which was associated with the drug combinations, but not the individual agents, increasing the protein levels of Beclin1 and ATG5. Knock down of Beclin1 or ATG5, or expression of an activated form of mTOR, significantly reduced uveal melanoma cell killing. GZ17-6.02 and neratinib interacted to enhance eIF2α S51 phosphorylation, i.e., its inactivation resulting in endoplasmic reticulum stress signaling. Knock down of eIF2α significantly reduced autophagosome formation, flux and uveal melanoma cell killing. Using a syngeneic immune-competent tumor model system, dosing of mice with GZ17-6.02 resulted in tumor uptake of all three components of the drug: curcumin, harmine and isovanillin.

The GZ17-6.02 combination with neratinib not only inactivated eIF2α but also reduced signaling by STAT3 and STAT5 and reduced the expression of BCL-XL and MCL1. Knock down of eIF2α prevented the drug-induced decline in BCL-XL and MCL1 levels. Expression of an activated form of STAT3 enhanced basal expression of BCL-XL and MCL1 and prevented the drugs from lowering their levels. Over-expression of BCL-XL or knock down of toxic BH3 domain proteins significantly reduced drug-induced tumor cell killing, however, expression of dominant negative caspase 9 was less protective than BCL-XL suggestive that both apoptotic and non-apoptotic cell killing was occurring.

Almost half of all uveal melanomas express a mutated inactive form of BAP1; mutation of a single allele of BAP1 is associated with tumor progression and metastatic spread [17, 31–33]. BAP1 is a deubiquitinase, regulating the ubiquitination of Histone 2A [33]. We found that GZ17-6.02 as a single agent increased BAP1 expression in uveal melanoma cells that was not altered by ERBB receptor inhibitors. In addition to histone ubiquitination, BAP1 also plays a role in regulating CpG site DNA methylation and histone methylation [21, 34–38]. The BAP1 promoter itself is subject to epigenetic regulation and hypermethylation of its DNA is inversely correlated with BAP1 mRNA expression. Methylation of the BAP1 promoter can be used as a proxy for its genomic copy loss and its reduced protein levels, i.e., the promoter of a mutated BAP1 allele is methylated inactive. Hence, our discovery that GZ17-6.02 is enhancing BAP1 expression favors increased expression from the wild type unmutated BAP1 allele in uveal melanoma cells.

GZ17-6.02 changed the methylation and acetylation of Histone H3 in uveal melanoma cells, most notably, the mono-methylation of lysine 9 and lysine 4 was reduced and this correlated with a trend for increased di-methylation of lysine 4. The acetylation of lysine 9 remained elevated for 48 h. Methylation of di- and tri-methylation of lysine 4 in histone H3 in uveal melanoma cells is believed to represent activation of transcription, on the other hand, demethylation of lysine 4 is associated with transcriptional repression [39–41]. The methylation of histone 3 lysine 9 was reduced by GZ17-6.02, which is of note because the methylation of lysine 9 regulates the survival response of cells to endoplasmic reticulum stress, with lysine 9 methylation being predictive of resistance to ER stress-induced killing [42]. In prostate cancer, methylation of lysine 9 drives androgen receptor antagonist resistance [43]. The histone-lysine methyltransferase G9a is often over-expressed in tumors and blocking its function, reducing lysine 9 methylation, reduces tumorigenic potential and enhances autophagic-induced cell death [44]. Others have linked ERBB1 signaling, lysine 9 acetylation in the ability of cells to activate ATM and initiate a DNA damage response [45].

The co-transcription factors YAP and TAZ (the Hippo pathway) play a central role in initiation, the development and therapeutic resistance of uveal melanoma cells [26–30]. Regulation of the Hippo pathway in uveal melanoma cells has been linked to signaling by the mutated G alpha proteins Gα_11_ and Gα_q_ [29, 30, 46]. We discovered that GZ17-6.02 as a single agent and more so when combined with neratinib enhanced the phosphorylation of YAP at S127 and S397 and TAZ at S89. Increased phosphorylation at these sites causes YAP and TAZ to exit the nucleus and S397 phosphorylation predisposes YAP to be degraded, as was observed in Mel4 cells. Other studies from our group in NSCLC and pancreatic cancer cells using neratinib, as well as prior work in uveal melanoma cells, have demonstrated that the drug rapidly reduces the protein levels of RAS proteins and mutant G alpha proteins, resulting in enhanced macroautophagy-dependent tumor cell killing [23, 47–49]. Studies beyond the scope of the present manuscript will be required to fully understand the regulation of BAP1 and the Hippo pathway in uveal melanoma cells.

## MATERIALS AND METHODS

### Materials

The PDX UM isolates cell lines were kindly provided by Dr. Kirkwood at the University of Pittsburgh. Afatinib and doxorubicin were purchased from Selleckchem (Houston, TX, USA). The established MP46 uveal melanoma cell line was obtained from the ATCC (Bethesda, MD, USA). Neratinib maleate was kindly provided by Puma Biotechnology (Los Angeles, CA, USA). All Materials were obtained as described in the references [1–9]. Trypsin-EDTA, DMEM, RPMI, penicillin-streptomycin were purchased from GIBCOBRL (GIBCOBRL Life Technologies, Grand Island, NY, USA). Other reagents and performance of experimental procedures were as described [1–9]. Antibodies were purchased from Cell Signaling Technology (Danvers, MA, USA); Abgent (San Diego, CA, USA); Novus Biologicals (Centennial, CO, USA); Abcam (Cambridge, UK); and Santa Cruz Biotechnology (Dallas, TX, USA). Specific multiple independent siRNAs to knock down the expression of CD95, FADD, Beclin1, ATG5, AMPKα_1_, ATM, BIM, BAX, BAK, BID and eIF2α, and scramble control, were purchased from Qiagen (Hilden, Germany) and Thermo Fisher (Waltham, MA, USA). Control studies were presented in prior manuscripts showing on-target specificity of our siRNAs, primary antibodies, and our phospho-specific antibodies to detect both total protein levels and phosphorylated levels of proteins [1–9] (Table 8).

**Table 8:** Control data for transfection efficiency of uveal melanoma cells

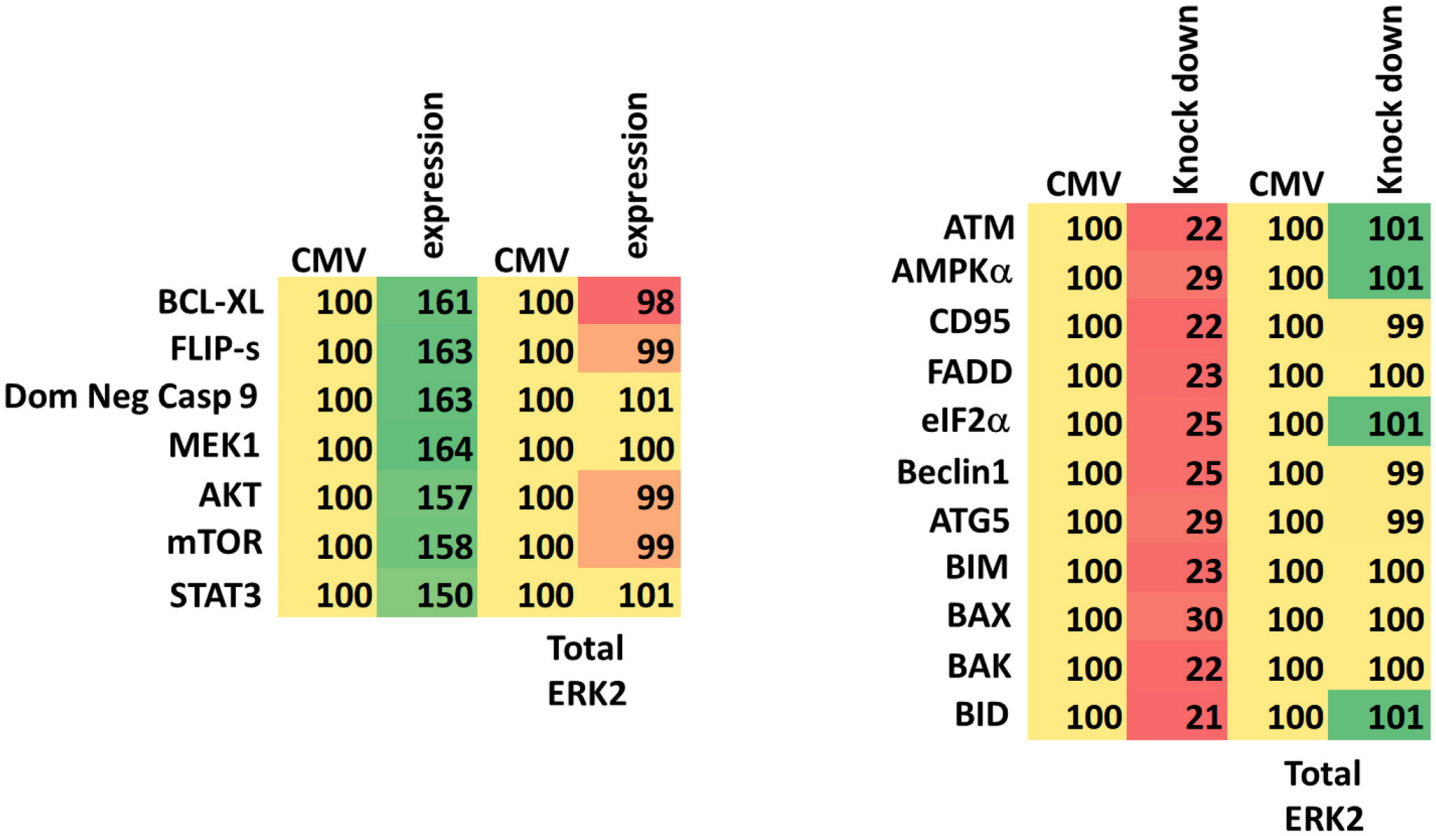

MEL2 cells as indicated were transfected with siRNA molecules to knock down the expression of the indicated proteins or transfected with plasmids to over-express the indicated proteins. The percentage remaining after knock-down or the percentage over-expression above basal levels is indicated. (*n **=*** 3 +/− SD) (total ERK2 is included as an invariant total protein loading control).

### METHODS

All bench-side Methods used in this manuscript have been previously performed and described in detail in the peer-reviewed references [1–9].

### Assessments of protein expression and protein phosphorylation [1–9]

At various time-points after the initiation of drug exposure, cells in 96-well plates are fixed in place using paraformaldehyde and using Triton X100 for permeabilization. Standard immunofluorescent blocking procedures are employed, followed by incubation of different wells with a variety of validated primary antibodies and subsequently validated fluorescent-tagged secondary antibodies are added to each well. Assessments of staining intensity were made using a Hermes wide field microscope (Idea Biotechnology, Rehovot, Israel).

### Detection of cell death by trypan blue assay [1–9]

Cells were treated with vehicle control or with drugs alone or in combination for 24 h. At the indicated time points cells were harvested by trypsinization and centrifugation. Cell pellets were resuspended in PBS and mixed with trypan blue agent. Viability was determined microscopically using a hemocytometer. Five hundred cells from randomly chosen fields were counted and the number of dead cells was counted and expressed as a percentage of the total number of cells counted.

### Transfection of cells with siRNA or with plasmids [1–9]

Cells were plated and 24 h after plating, transfected. Plasmids to express FLIP-s, BCL-XL, dominant negative caspase 9, activated AKT, activated STAT3, activated mTOR and activated MEK1 EE were used throughout the study (Addgene, Waltham, MA). Empty vector plasmid (CMV) was used as a control. For siRNA transfection, 10 nM of the annealed siRNA or the negative control (a “scrambled” sequence with no significant homology to any known gene sequences from mouse, rat or human cell lines) were used.

### Assessments of autophagosome and autolysosome levels [1–9, 50]

Cells were transfected with a plasmid to express LC3-GFP-RFP (Addgene, Watertown MA). Twenty-four hours after transfection, cells are treated with vehicle control or the indicated drugs alone or in combination. Cells were randomly imaged and recorded at 60X magnification on a Ziess microscope 4 h and 8 h after drug exposure. The mean number of intense fluorescing (GFP+RFP+) and (RFP+) puncta per cell was determined from >50 randomly selected cells per condition.

### Treatment of mice with GZ17-6.02 and measuring the levels of curcumin, harmine and isovanillin in pre-formed flank tumors

Female BALB/c mice were implanted with syngeneic CT26 colon cancer cells which express a mutant KRAS. Tumors were permitted to form for 14 days where tumor volumes were ~100 mm^3^. Mice were treated daily with either vehicle control or with GZ17-6.02 (50 mg/kg). After 30 days, tumors were isolated, and flash frozen in liquid N_2_. Tumor material was cryo-crushed under liquid nitrogen, then homogenized in glass tube/rotary pestle with 3 parts water. Curcumin and harmine were analyzed together by extraction of tumor homogenate with ethylacetate, nitrogen stream evaporation, and reconstitution by mobile phase A-acetonitrile (1:1). Isovaniline was extracted from tumor homogenate by chloroform-isopropanol (4:1), organic phase dried by nitrogen stream, and the residue derivatized by pentafluorobenzoyl-hydroxylamine (PFBHA) in water-methanol-acetonitrile (1:1.5:2.5) with 40 mM ammonium acetate at pH 4. The obtained samples were analyzed by LC/MS/MS (Agilent/AB-SCIEX API5500). Curcumin-d6, harmine-d3, and isovanilin-d3 were used as internal standards. Lower limits of quantification for curcumin, harmine, and isovanilin in tumor samples were 1.6, 1.6, and 20 ng/g wet tissue, respectively.

### Data analysis

Comparison of the effects of various treatments was using one-way ANOVA for normalcy followed by a two tailed Student’s *t*-test with multiple comparisons. Differences with a *p*-value of < 0.05 were considered statistically significant. Experiments are the means of multiple individual data points per experiment from 3 independent experiments (± SD).

## Abbreviations

ERK: extracellular regulated kinase
PI3K: phosphatidyl inositol 3 kinase
ca: constitutively active
dn: dominant negative
ER: endoplasmic reticulum
AMPK: AMP-dependent protein kinase
mTOR: mammalian target of rapamycin
JAK: Janus Kinase
STAT: Signal Transducers and Activators of Transcription
MAPK: mitogen activated protein kinase
PTEN: phosphatase and tensin homologue on chromosome ten
ROS: reactive oxygen species
CMV: empty vector plasmid
si: small interfering
SCR: scrambled
VEH: vehicle
602: GZ17-6.02
AFAT: afatinib
NER: neratinib
MHCA: major histocompatibility class A

## References

[1] Booth L , Roberts JL , West C , Von Hoff D , Dent P . GZ17-6.02 initiates DNA damage causing autophagosome-dependent HDAC degradation resulting in enhanced anti-PD1 checkpoint inhibitory antibody efficacy. J Cell Physiol. 2020; 235:8098–113. 10.1002/jcp.29464. 31951027

[2] Booth L , West C , Hoff DV , Dent P . GZ17-6.02 and Doxorubicin Interact to Kill Sarcoma Cells via Autophagy and Death Receptor Signaling. Front Oncol. 2020; 10:1331. 10.3389/fonc.2020.01331. 32983965 PMC7492267

[3] Booth L , West C , Von Hoff D , Kirkwood JM , Dent P . GZ17-6.02 Interacts With [MEK1/2 and B-RAF Inhibitors] to Kill Melanoma Cells. Front Oncol. 2021; 11:656453. 10.3389/fonc.2021.656453. 33898322 PMC8061416

[4] Booth L , West C , Moore RP , Von Hoff D , Dent P . GZ17-6.02 and Pemetrexed Interact to Kill Osimertinib-Resistant NSCLC Cells That Express Mutant ERBB1 Proteins. Front Oncol. 2021; 11:711043. 10.3389/fonc.2021.711043. 34490108 PMC8417372

[5] Booth L , West C , Moore RP , Von Hoff D , Dent P . GZ17-6.02 and palbociclib interact to kill ER+ breast cancer cells. Oncotarget. 2022; 13:92–104. 10.18632/oncotarget.28177. 35035775 PMC8754587

[6] Booth L , West C , Moore RP , Hoff DV , Dent P . GZ17-6.02 and axitinib interact to kill renal carcinoma cells. Oncotarget. 2022; 13:281–90. 10.18632/oncotarget.28189. 35136485 PMC8815785

[7] Booth L , West C , Von Hoff D , Dent P . Mechanisms of GZ17-6.02 resistance. Anticancer Drugs. 2022; 33:415–23. 10.1097/CAD.0000000000001203. 35276694

[8] Booth L , Roberts JL , West C , Dent P . GZ17-6.02 kills prostate cancer cells *in vitro* and *in vivo* . Front Oncol. 2022; 12:1045459. 10.3389/fonc.2022.1045459. 36408163 PMC9671078

[9] Bordeaux ZA , Kwatra SG , Booth L , Dent P . A novel combination of isovanillin, curcumin, and harmine (GZ17-6.02) enhances cell death and alters signaling in actinic keratoses cells when compared to individual components and two-component combinations. Anticancer Drugs. 2023; 34:544–50. 10.1097/CAD.0000000000001425. 36847046

[10] Bai H , Bosch JJ , Heindl LM . Current management of uveal melanoma: A review. Clin Exp Ophthalmol. 2023; 51:484–94. 10.1111/ceo.14214. 37076276

[11] Howlett S , Carter TJ , Shaw HM , Nathan PD . Tebentafusp: a first-in-class treatment for metastatic uveal melanoma. Ther Adv Med Oncol. 2023; 15:17588359231160140. 10.1177/17588359231160140. 36970111 PMC10031621

[12] O’Hayre M , Degese MS , Gutkind JS . Novel insights into G protein and G protein-coupled receptor signaling in cancer. Curr Opin Cell Biol. 2014; 27:126–35. 10.1016/j.ceb.2014.01.005. 24508914 PMC4021379

[13] Van Raamsdonk CD , Bezrookove V , Green G , Bauer J , Gaugler L , O’Brien JM , Simpson EM , Barsh GS , Bastian BC . Frequent somatic mutations of GNAQ in uveal melanoma and blue naevi. Nature. 2009; 457:599–602. 10.1038/nature07586. 19078957 PMC2696133

[14] Van Raamsdonk CD , Griewank KG , Crosby MB , Garrido MC , Vemula S , Wiesner T , Obenauf AC , Wackernagel W , Green G , Bouvier N , Sozen MM , Baimukanova G , Roy R , et al. Mutations in GNA11 in uveal melanoma. N Engl J Med. 2010; 363:2191–99. 10.1056/NEJMoa1000584. 21083380 PMC3107972

[15] Koopmans AE , Vaarwater J , Paridaens D , Naus NC , Kilic E , de Klein A , and Rotterdam Ocular Melanoma Study group. Patient survival in uveal melanoma is not affected by oncogenic mutations in GNAQ and GNA11. Br J Cancer. 2013; 109:493–96. 10.1038/bjc.2013.299. 23778528 PMC3721402

[16] Onken MD , Worley LA , Long MD , Duan S , Council ML , Bowcock AM , Harbour JW . Oncogenic mutations in GNAQ occur early in uveal melanoma. Invest Ophthalmol Vis Sci. 2008; 49:5230–34. 10.1167/iovs.08-2145. 18719078 PMC2634606

[17] Harbour JW , Onken MD , Roberson ED , Duan S , Cao L , Worley LA , Council ML , Matatall KA , Helms C , Bowcock AM . Frequent mutation of BAP1 in metastasizing uveal melanomas. Science. 2010; 330:1410–13. 10.1126/science.1194472. 21051595 PMC3087380

[18] Piaggio F , Croce M , Reggiani F , Monti P , Bernardi C , Ambrosio M , Banelli B , Dogrusöz M , Jockers R , Bordo D , Puzone R , Viaggi S , Coviello D , et al. In uveal melanoma Gα-protein GNA11 mutations convey a shorter disease-specific survival and are more strongly associated with loss of BAP1 and chromosomal alterations than Gα-protein GNAQ mutations. Eur J Cancer. 2022; 170:27–41. 10.1016/j.ejca.2022.04.013. 35580369

[19] Robertson AG , Shih J , Yau C , Gibb EA , Oba J , Mungall KL , Hess JM , Uzunangelov V , Walter V , Danilova L , Lichtenberg TM , Kucherlapati M , Kimes PK , et al, and TCGA Research Network. Integrative Analysis Identifies Four Molecular and Clinical Subsets in Uveal Melanoma. Cancer Cell. 2017; 32:204–20.e15. 10.1016/j.ccell.2017.07.003. 28810145 PMC5619925

[20] van de Nes JA , Nelles J , Kreis S , Metz CH , Hager T , Lohmann DR , Zeschnigk M . Comparing the Prognostic Value of BAP1 Mutation Pattern, Chromosome 3 Status, and BAP1 Immunohistochemistry in Uveal Melanoma. Am J Surg Pathol. 2016; 40:796–805. 10.1097/PAS.0000000000000645. 27015033

[21] Hoffmann F , Fröhlich A , Sirokay J , de Vos L , Zarbl R , Dietrich J , Strieth S , Landsberg J , Dietrich D . DNA methylation of GITR, OX40, 4-1BB, CD27, and CD40 correlates with BAP1 aberrancy and prognosis in uveal melanoma. Melanoma Res. 2023; 33:116–25. 10.1097/CMR.0000000000000879. 36735464

[22] Ferrier ST , Burnier JV . Novel Methylation Patterns Predict Outcome in Uveal Melanoma. Life (Basel). 2020; 10:248. 10.3390/life10100248. 33092094 PMC7589184

[23] Booth L , Roberts JL , Sander C , Lalani AS , Kirkwood JM , Hancock JF , Poklepovic A , Dent P . Neratinib and entinostat combine to rapidly reduce the expression of K-RAS, N-RAS, Gα_q_ and Gα_11_ and kill uveal melanoma cells. Cancer Biol Ther. 2019; 20:700–10. 10.1080/15384047.2018.1551747. 30571927 PMC6606002

[24] Bellese G , Tagliatti E , Gagliani MC , Santamaria S , Arnaldi P , Falletta P , Rusmini P , Matteoli M , Castagnola P , Cortese K . Neratinib is a TFEB and TFE3 activator that potentiates autophagy and unbalances energy metabolism in ERBB2+ breast cancer cells. Biochem Pharmacol. 2023; 213:115633. 10.1016/j.bcp.2023.115633. 37269887

[25] Santamaria S , Gagliani MC , Bellese G , Marconi S , Lechiara A , Dameri M , Aiello C , Tagliatti E , Castagnola P , Cortese K . Imaging of Endocytic Trafficking and Extracellular Vesicles Released Under Neratinib Treatment in ERBB2^+^ Breast Cancer Cells. J Histochem Cytochem. 2021; 69:461–73. 10.1369/00221554211026297. 34126793 PMC8246527

[26] Brouwer NJ , Konstantinou EK , Gragoudas ES , Marinkovic M , Luyten GPM , Kim IK , Jager MJ , Vavvas DG . Targeting the YAP/TAZ Pathway in Uveal and Conjunctival Melanoma With Verteporfin. Invest Ophthalmol Vis Sci. 2021; 62:3. 10.1167/iovs.62.4.3. 33798262 PMC8024781

[27] Vader MJC , Madigan MC , Versluis M , Suleiman HM , Gezgin G , Gruis NA , Out-Luiting JJ , Bergman W , Verdijk RM , Jager MJ , van der Velden PA . GNAQ and GNA11 mutations and downstream YAP activation in choroidal nevi. Br J Cancer. 2017; 117:884–87. 10.1038/bjc.2017.259. 28809862 PMC5590000

[28] Li H , Li Q , Dang K , Ma S , Cotton JL , Yang S , Zhu LJ , Deng AC , Ip YT , Johnson RL , Wu X , Punzo C , Mao J . YAP/TAZ Activation Drives Uveal Melanoma Initiation and Progression. Cell Rep. 2019; 29:3200–11.e4. 10.1016/j.celrep.2019.03.021. 31801083 PMC7871510

[29] Feng X , Arang N , Rigiracciolo DC , Lee JS , Yeerna H , Wang Z , Lubrano S , Kishore A , Pachter JA , König GM , Maggiolini M , Kostenis E , Schlaepfer DD , et al. A Platform of Synthetic Lethal Gene Interaction Networks Reveals that the GNAQ Uveal Melanoma Oncogene Controls the Hippo Pathway through FAK. Cancer Cell. 2019; 35:457–72.e5. 10.1016/j.ccell.2019.01.009. 30773340 PMC6737937

[30] Yu FX , Luo J , Mo JS , Liu G , Kim YC , Meng Z , Zhao L , Peyman G , Ouyang H , Jiang W , Zhao J , Chen X , Zhang L , et al. Mutant Gq/11 promote uveal melanoma tumorigenesis by activating YAP. Cancer Cell. 2014; 25:822–30. 10.1016/j.ccr.2014.04.017. 24882516 PMC4075337

[31] Kwon J , Lee D , Lee SA . BAP1 as a guardian of genome stability: implications in human cancer. Exp Mol Med. 2023; 55:745–54. 10.1038/s12276-023-00979-1. 37009801 PMC10167335

[32] Masclef L , Ahmed O , Estavoyer B , Larrivée B , Labrecque N , Nijnik A , Affar EB . Roles and mechanisms of BAP1 deubiquitinase in tumor suppression. Cell Death Differ. 2021; 28:606–25. 10.1038/s41418-020-00709-4. 33462414 PMC7862696

[33] Jager MJ , Shields CL , Cebulla CM , Abdel-Rahman MH , Grossniklaus HE , Stern MH , Carvajal RD , Belfort RN , Jia R , Shields JA , Damato BE . Uveal melanoma. Nat Rev Dis Primers. 2020; 6:24. 10.1038/s41572-020-0158-0. 32273508

[34] Walpole S , Pritchard AL , Cebulla CM , Pilarski R , Stautberg M , Davidorf FH , de la Fouchardière A , Cabaret O , Golmard L , Stoppa-Lyonnet D , Garfield E , Njauw CN , Cheung M , et al. Comprehensive Study of the Clinical Phenotype of Germline BAP1 Variant-Carrying Families Worldwide. J Natl Cancer Inst. 2018; 110:1328–41. 10.1093/jnci/djy171. 30517737 PMC6292796

[35] Scheuermann JC , de Ayala Alonso AG , Oktaba K , Ly-Hartig N , McGinty RK , Fraterman S , Wilm M , Muir TW , Müller J . Histone H2A deubiquitinase activity of the Polycomb repressive complex PR-DUB. Nature. 2010; 465:243–47. 10.1038/nature08966. 20436459 PMC3182123

[36] Bakhoum MF , Curtis EJ , Goldbaum MH , Mischel PS . BAP1 methylation: a prognostic marker of uveal melanoma metastasis. NPJ Precis Oncol. 2021; 5:89. 10.1038/s41698-021-00226-8. 34593944 PMC8484429

[37] LaFave LM , Béguelin W , Koche R , Teater M , Spitzer B , Chramiec A , Papalexi E , Keller MD , Hricik T , Konstantinoff K , Micol JB , Durham B , Knutson SK , et al. Loss of BAP1 function leads to EZH2-dependent transformation. Nat Med. 2015; 21:1344–49. 10.1038/nm.3947. 26437366 PMC4636469

[38] Kotake Y , Cao R , Viatour P , Sage J , Zhang Y , Xiong Y . pRB family proteins are required for H3K27 trimethylation and Polycomb repression complexes binding to and silencing p16INK4alpha tumor suppressor gene. Genes Dev. 2007; 21:49–54. 10.1101/gad.1499407. 17210787 PMC1759899

[39] Leadem BR , Kagiampakis I , Wilson C , Cheung TK , Arnott D , Trojer P , Classon M , Easwaran H , Baylin SB . A KDM5 Inhibitor Increases Global H3K4 Trimethylation Occupancy and Enhances the Biological Efficacy of 5-Aza-2’-Deoxycytidine. Cancer Res. 2018; 78:1127–39. 10.1158/0008-5472.CAN-17-1453. 29282222 PMC5935520

[40] Zhang H , Liu X , Chen Y , Xu R , He S . KDOAM-25 Overcomes Resistance to MEK Inhibitors by Targeting KDM5B in Uveal Melanoma. Biomed Res Int. 2022; 2022:1556485. 10.1155/2022/1556485. . Retraction in: Biomed Res Int. 2023; 2023:9857986. 10.1155/2022/1556485. 36212716 PMC9534647

[41] Jain K , Marunde MR , Burg JM , Gloor SL , Joseph FM , Poncha KF , Gillespie ZB , Rodriguez KL , Popova IK , Hall NW , Vaidya A , Howard SA , Taylor HF , et al. An acetylation-mediated chromatin switch governs H3K4 methylation read-write capability. Elife. 2023; 12:e82596. 10.7554/eLife.82596. 37204295 PMC10229121

[42] Diaz-Bulnes P , Saiz ML , Corte-Iglesias V , Rodrigues-Diez RR , Bernardo Florez A , Ruiz Bernet C , Martin Martin C , Ruiz-Ortega M , Suarez-Alvarez B , López-Larrea C . Demethylation of H3K9 and H3K27 Contributes to the Tubular Renal Damage Triggered by Endoplasmic Reticulum Stress. Antioxidants (Basel). 2022; 11:1355. 10.3390/antiox11071355. 35883846 PMC9312208

[43] Baratchian M , Tiwari R , Khalighi S , Chakravarthy A , Yuan W , Berk M , Li J , Guerinot A , de Bono J , Makarov V , Chan TA , Silverman RH , Stark GR , et al. H3K9 methylation drives resistance to androgen receptor-antagonist therapy in prostate cancer. Proc Natl Acad Sci U S A. 2022; 119:e2114324119. 10.1073/pnas.2114324119. 35584120 PMC9173765

[44] Thng DKH , Hooi L , Toh CCM , Lim JJ , Rajagopalan D , Syariff IQC , Tan ZM , Rashid MBMA , Zhou L , Kow AWC , Bonney GK , Goh BKP , Kam JH , et al. Histone-lysine N-methyltransferase EHMT2 (G9a) inhibition mitigates tumorigenicity in Myc-driven liver cancer. Mol Oncol. 2023; 17:2275–94. 10.1002/1878-0261.13417. 36896891 PMC10620125

[45] Dittmann K , Mayer C , Rodemann HP , Huber SM . EGFR cooperates with glucose transporter SGLT1 to enable chromatin remodeling in response to ionizing radiation. Radiother Oncol. 2013; 107:247–51. 10.1016/j.radonc.2013.03.016. 23602371

[46] Feng X , Chen Q , Gutkind JS . Oncotargeting G proteins: The Hippo in the room. Oncotarget. 2014; 5:10997–99. 10.18632/oncotarget.2815. 25526026 PMC4294369

[47] Dent P , Booth L , Poklepovic A , Von Hoff D , Martinez J , Zhou Y , Hancock JF . Osimertinib-resistant NSCLC cells activate ERBB2 and YAP/TAZ and are killed by neratinib. Biochem Pharmacol. 2021; 190:114642. 10.1016/j.bcp.2021.114642. 34077739 PMC11082938

[48] Dent P , Booth L , Poklepovic A , Martinez J , Hoff DV , Hancock JF . Neratinib degrades MST4 via autophagy that reduces membrane stiffness and is essential for the inactivation of PI3K, ERK1/2, and YAP/TAZ signaling. J Cell Physiol. 2020; 235:7889–99. 10.1002/jcp.29443. 31912905 PMC10324541

[49] Dent P , Booth L , Roberts JL , Liu J , Poklepovic A , Lalani AS , Tuveson D , Martinez J , Hancock JF . Neratinib inhibits Hippo/YAP signaling, reduces mutant K-RAS expression, and kills pancreatic and blood cancer cells. Oncogene. 2019; 38:5890–904. 10.1038/s41388-019-0849-8. 31253872 PMC7133220

[50] Klionsky DJ , Abdel-Aziz AK , Abdelfatah S , Abdellatif M , Abdoli A , Abel S , Abeliovich H , Abildgaard MH , Abudu YP , Acevedo-Arozena A , Adamopoulos IE , Adeli K , Adolph TE , et al. Guidelines for the use and interpretation of assays for monitoring autophagy (4th edition)^1^. Autophagy. 2021; 17:1–382. 10.1080/15548627.2020.1797280. 33634751 PMC7996087

